# Moderate physical activity from childhood contributes to metabolic health and reduces hepatic fat accumulation in adult rats

**DOI:** 10.1186/1476-511X-12-29

**Published:** 2013-03-06

**Authors:** Leandro Pereira de Moura, Amanda Christine da Silva Sponton, Michel Barbosa de Araújo, Rodrigo Augusto Dalia, José Rodrigo Pauli, Maria Alice Rostom de Mello

**Affiliations:** 1 UNESP/Rio Claro, Bioscience Institute, Physical Education Department, São Paulo State University, Rio Claro, Brazil; 2 UNICAMP/Limeira, Applied Sciences Faculty, Sports Science Course, Campinas State University, Campinas, Brazil; 3 Department of Physical Education, Universidade Estadual Paulista (UNESP), Avenida 24ª n° 1515, Bela Vista, P.O. Box 199, 13506-900, Rio Claro, SP, Brazil

**Keywords:** Moderate exercise, Metabolic health, Hepatic fat accumulation

## Abstract

**Background:**

Obesity, oxidative stress and inflammation, by triggering insulin resistance, may contribute to the accumulation of hepatic fat, and this accumulation by lipotoxicity can lead the organ to fail. Because obesity is growing at an alarming rate and, worryingly, in a precocious way, the present study aimed to investigate the effects of moderate physical training performed from childhood to adulthood on liver fat metabolism in rats.

**Methods:**

Twenty rats that were 28 days old were divided into two groups: control (C) and trained (T). The C Group was kept in cages without exercise, and the T group was submitted to swimming exercise for 1 hour/day, 5 days/week from 28 to 90 days of age (8 weeks) at 80% of the anaerobic threshold determined by the lactate minimum test. At the end of the experiment, the body weight gain, insulin sensitivity (glucose disappearance rate during the insulin tolerance test), concentrations of free fatty acids (FFA) and triglycerides (TG) and hepatic lipogenic rate were analyzed. For the statistical analysis, the Student t-test was used with the level of significance preset at 5%.

**Results:**

The T group showed lower body weight gain, FFA concentrations, fat accumulation, hepatic lipogenic rate and insulin resistance.

**Conclusion:**

The regular practice of moderate physical exercise from childhood can contribute to the reduction of obesity and insulin resistance and help prevent the development of accumulation of hepatic fat in adulthood.

## Background

Obesity is a major public health problem and is a risk factor for the development of many fatal diseases, particularly type 2 diabetes and nonalcoholic fatty liver disease (NAFLD) [1-4]. The prevalence of NAFLD in obese individuals increases significantly, reaching 50-75% [5]. The increase in the prevalence of NAFLD is presumed to parallel the increased prevalence of obesity and diabetes in all age groups.

Described by Ludwig and colleagues in 1980 [6], NAFLD is clinically characterized by the deposition of fat droplets in liver hepatocytes [5,7] and varies from simple macrovesicular steatosis to steatohepatitis, advanced fibrosis and cirrhosis. NAFLD may be the main cause of morbidity and mortality related to liver diseases, with the potential to progress to liver failure. However, it should be noted that the progression to fibrosis or cirrhosis appears to occur only in patients with evidence of steatohepatitis.

The etiopathogenesis of NAFLD includes oxidative stress, inflammation and insulin resistance and a consequently increased synthesis of triglycerides and retention in the hepatocyte, leading to macrovesicular steatosis. Mitochondrial dysfunction also assumes an important role, leading to lower lipid oxidation and contributing to fat accumulation and injury in hepatocytes. The attack of the organ also involves oxidative stress, causing peroxidation of membrane lipids in the hepatocytes and cytokine production, especially of tumor necrosis factor alpha (TNF-α), which are partly responsible for the progression of hepatic steatosis to steatohepatitis and cirrhosis [5,7].

Currently, there are no drugs to treat this disease, and decreased fat intake and regular exercise are recommended for people diagnosed with NAFLD [8,9]. The change in lifestyle should occur as soon as possible because obesity in childhood and adolescence is a major risk factor for obesity and its co-morbidities in adulthood, including NAFLD [10,11]. Therefore, investigating whether physical exercise during childhood can prevent the development of obesity and NAFLD and determining the appropriate intensity become important.

Due to the limitations of human studies, lab animals are commonly used in scientific research, and the ability to control the intensity of exercise performed by these animals in adulthood has been extensively investigated. However, there are special situations, such as weanling rats that simulate children, that have not yet been elucidated in the literature. Insufficient information is also available on the effects induced by moderate exercise performed from childhood to adulthood. Therefore, this study aimed to investigate the effects of moderate exercise training (80% of the individual anaerobic threshold) performed from childhood to adulthood on liver fat metabolism in rats.

## Results

**Figure 1:**
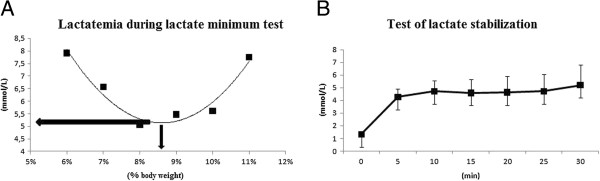
**A. Determination of the blood lactate threshold (BLT) from a single rat during the incremental swimming test. **Each dot indicates the blood lactate concentration (mmol/L) on the ordinate and the work overload on the abscissa. The BLT and workload were calculated with a second-order polynomial curve using Microsoft Excel®. The vertical arrow estimates the workload, and the horizontal arrow estimates the blood lactate concentration. **B**. Peripheral lactate concentration during exercise with a constant overload, which was estimated by BLT using the minimum blood lactate test. The results are expressed as the means ± standard deviation of 10 animals. * = different from the baseline (ANOVA, p <0.05).

The aerobic capacity for the weanling rats could be determined. The average minimum concentration of lactate (mmol/L) was 5.03 ± 1.15, and that obtained in the overload (% bw) was 8.59 ± 1.09 (Figure 1A). During a 30 minute exercise session with the animals supporting 100% of the overhead determined by the lactate minimum test, there was stabilization of the peripheral lactate concentration between 10 and 30 min of exercise (Figure 1B).

**Figure 2:**
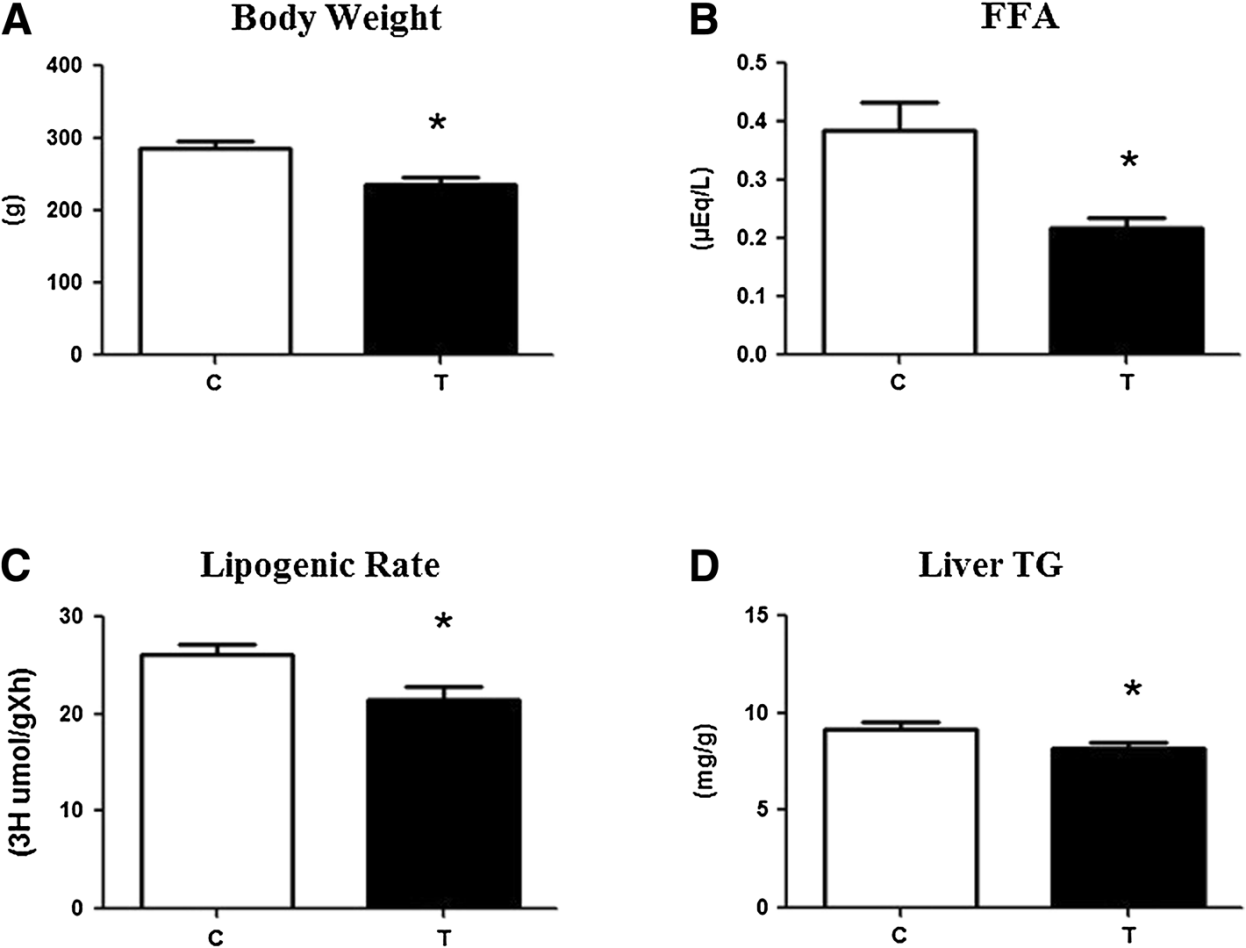
**The data on body weight, free fatty acid (FFA) level, liver lipogenic rate and liver triglyceride level, respectively, at the end of the experiment. **C = control, T = trained. * ≠ T group compared with C. The data represent the means ± standard deviation (n = 10 animals/group).

The animals from the T group showed lower weight gain, free fatty acid and triglycerides concentrations and hepatic lipogenic rate compared with the sedentary group (Figure 2A, B, C and D).

**Figure 3:**
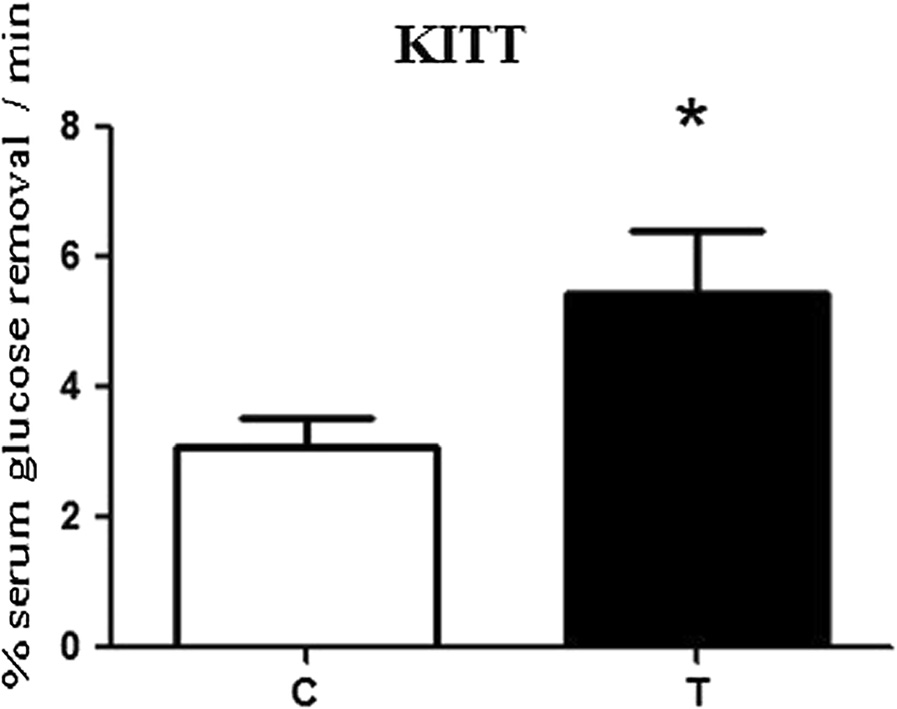
**Glucose removal rate after the insulin tolerance test calculated by KITT. **C = control, T = trained. * ≠ T group when compared with C. The data are represented as the means ± standard deviation (n = 10 animals/group).

The T group animals showed a higher uptake of glucose per minute as measured by the insulin tolerance test (ITT). These animals were more sensitive to insulin than the sedentary group animals (Figure 3).

## Discussion

Cases of liver carcinomas are the fifth most common in the global ranking of cancer diagnoses [12], growing in line with the growth of NAFLD [13,14]. Because the latter disease may be due to the insulin resistance found in obese individuals and because the incidence of obesity has been increasing worldwide, affecting children, youths and adult men and women [15], the importance of the prevention and treatment of obesity, which can be initiated in childhood by physical exercise, is important to minimize the inconvenience caused by this condition in adulthood.

Because the current findings surrounding the effect of moderate physical exercise (80% of Lan) on the development of NAFLD are not clear, the present study examined the effect of a protocol of moderate training (80% of individual aerobic capacity) from childhood to adulthood on fat metabolism in the liver.

A thorough knowledge of the appropriate exercise intensity is of paramount importance. There are numerous studies that show different responses of an organism to different intensities of exercise [16-18]. According to Dubé et al. [19], there is a high correlation between the dose/response of physical exercise intensity with insulin sensitivity, with the recovery of individuals after stroke [20] or acute myocardial infarction [21] and with fibromyalgia [22]. These authors also showed that the higher the intensity of exercise, the better the body's response to that exercise. Therefore, a precise determination of the individual aerobic capacity prior to start of a physical training program is important.

To determine the aerobic capacity, the maximum lactate steady state (MLSS) test is considered the gold standard, but it requires several days of tests, and its application can be limited in some special cases. Therefore, in 1993, Tegtbur et al. [23] developed a test to determine the anaerobic threshold (AT) called the lactate minimum test, which has been considered a method to determine the exercise intensity corresponding to the equilibrium between the production and removal of blood lactate [23,24] or MLSS [25,26]. In addition to this protocol being considered a valid method to determine aerobic capacity, it also enables the identification of the intensity corresponding to the MLSS in a single test session [25,26].

Moreover, studies of the metabolic effects of exercise in rats are often challenged by a lack of information about the intensity of the effort performed by the animal during exercise. Therefore, Gobatto et al. [27] adapted the MLSS test from human adults to Wistar rats, and after understanding that human tests could be adapted to animal models, Araujo et al. [28] adapted the lactate minimum test for adult Wistar rats in swimming exercise. These authors suggested that regardless of the overload in the test, the minimum blood lactate concentration was approximately 5 mmol/L. Because the application of overloads to rats during exercise in the water is relative to body weight, these tests may appear inappropriate for rats that are too light or in an accelerated growth phase, such as weanling rats. However, this study successfully determined the AT in weanling rats, which was previously unknown in the literature. This study also confirmed the data previously described by Gobatto et al. [27] and Araújo et al. [28], which showed that the lactate concentration at AT, regardless of the intensity obtained, was approximately 5 mmol/L.

After standardization of the lactate minimum test because the AT is often assumed to be characterized by a higher intensity of exercise in which the blood lactate does not oscillate more than 1 mmol/L from 10 to 30 min of exercise with a constant overload [29], four days after the lactate minimum test, the rats were submitted to exercise with constant overloads corresponding to those found in the test. The lactate behavior was similar to that found in the MLSS test, and thus the lactate minimum test can be an important tool to determine the AT for weanling rats in a single test.

The exercise intensity in cases of obesity and fat accumulation in the liver is a matter that has much been discussed currently because if the sustained intensity is high, there is a high fat oxidation during the exercise session. However, to restructure the organization for the future to sustain wear, this oxidation is increased after this practice, a condition known as excess post-exercise oxygen consumption (EPOC) in which the consumption is directly related to the intensity supported during the physical exertion; thus, the greater the exercise intensity, the greater the oxygen consumption as long as this condition persists [30-32].

When exercise is performed with the intensity ranging from mild to moderate, during which the energy demand is not required quickly, there will be time enough to metabolize the fatty acids (FA) used to feed this energy demand, and thus there will be a greater activation of the enzyme lipoprotein lipase. In turn, there will be an increase in the rate of lipolysis, and the FAs from adipose tissue will predominantly be oxidized to sustain exercise for a long time, causing a consequent decrease in fat mass and fat intake by the liver. This area of work is known as Fatmax [32,33].

In 2010, Da Silva et al. [34] showed that the intensity of exercise performed at 70% of AT decreased insulin resistance due to a decrease in inflammatory processes and also showed that rats swimming freely, without an overload but for a long period (6 hours), had improvements in insulin sensitivity. These results indicated that very light exercise can be compensated for by increasing the volume of exercise. However, this high volume training may not be able to be supported by obese individuals, especially in those activities that require the body weight to be supported, indicating that training protocols with shorter durations are important.

A study performed by Lima et al. [35], in which the effects of different exercise intensities in type 2 diabetic subjects were analyzed, revealed that diabetic patients who consumed high carbohydrate solutions oxidized even more fat compared with healthy subjects after high-intensity exercise (90% Lan). This protocol can improve insulin sensitivity and minimize the damage caused by insulin resistance in obese individuals.

If the exercise intensity is not known, the exercise can act as a villain in combating obesity. According to Mayer et al. [36], when exercise is performed at a very low intensity that is not accompanied by a decrease in hunger, the body mass can increase. According to these authors, the effect of physical exercise on satiety occurs at intense levels, in which elevated levels of catecholamine suppress the feeling of hunger. In this study, subjecting the animals to a moderate-intensity exercise (80% of AT) caused these animals to have a lower weight gain, leading to a lower amount of adipose tissue and thereby reducing the intake of fat by the liver, ultimately preventing the development of NAFLD.

The stored energy in the form of fat can act in a toxic way on the liver and lead to lipotoxicity of the liver [37] and even organ failure by activating apoptotic pathways [38]. Analyzing the accumulation of hepatic fat through the lipogenic rate and triacylglycerol concentrations in the tissue, the insulin resistance was shown to generate a favorable environment for the greater accumulation of hepatic fat, which can be explained by the fact the activation of the lipogenic transcription factor hepatic SREBP (sterol regulatory element-binding proteins) by high concentrations of insulin [39-41]. The state of hyperinsulinemia increases the activity of SREBP in the liver, thereby increasing the levels of lipogenic genes, such as fatty acid synthase (FAS), acetyl-CoA carboxylase (ACC) and Stearoyl-CoA Desaturase1 (SCD1) contributing to the buildup of hepatic fat. Recently, Cintra et al. [42] showed in mice fed a high-fat diet that regular physical exercise that reduces SREBP levels was also effective in reducing the levels of FAS and SCD1 and increasing the levels of ACC, thereby contributing to the reversal of hepatic steatosis.

Because unsaturated fatty acids can activate proinflammatory pathways through membrane receptors, such as the toll-like receptors (TLRs) [43], reducing the supply of lipid substrates as a result of the increased resistance to insulin-shaped fatty acids is necessary. In this study, moderate physical exercise, by promoting a decrease in concentrations of FFA, was effective in improving insulin sensitivity, which may be associated with increased insulin activity in insulin-sensitive tissues. According to Boden & Shulman [44], the responsiveness of peripheral tissues to insulin may be damaged due to an increase in the concentration of FFA, affecting the insulin signaling pathway and thus leading to type 2 diabetes. Another factor that may have contributed to the best response of the peripheral tissues to insulin was the strong power of muscle contraction, which increases the expression of proteins that participate in the insulin signaling pathway, causing glucose uptake through mechanisms independent of intracellular insulin in rats and mice, whether they are obese or lean, during one or more physical exercise sessions, thereby making the body less glucose-intolerant and insulin-dependent [42-49].

## Conclusion

According to our findings, the regular practice of moderate physical exercise beginning in childhood can contribute to the reduction of obesity and insulin resistance and help prevent the development of NAFLD in adulthood.

## Materials and methods

### Animals

This study included 30 weanling Wistar rats, 21 days old at the beginning of the experiment, from the Central Animal Facility of the Universidade Estadual Paulista (UNESP), Botucatu/SP, Brazil. The animals were housed in polyethylene cages measuring 37 cm × 31 cm × 16 cm (four rats per cage) and kept under a room temperature of 21°C and photoperiod of 12 hours light/dark. The rats were fed with standard Purina feed and water "ad libitum".

All animal experiments were performed in accordance with the Brazilian law for the scientific use of animals (Law No 11.794, of October 8, 2008). The protocols were approved by the Ethics Committee on the Use of Animals (CEUA) from the Institute of Biosciences, UNESP - Campus de Rio Claro (Protocol: 4573/2010).

### Experimental groups

The rats were randomized, from 21 to 90 days old, into two groups: sedentary (S) rats that remained inactive and did not perform physical exercise and trained (T) rats that were subjected to an exercise protocol of swimming at a moderate physical intensity of 80% of the individual anaerobic threshold (AT).

### Adaptation to water

All animals in group T were adapted to the water environment. The adaptation consisted of keeping the animal in shallow water at a temperature of 31 ± 1°C and thereby minimizing the stress experienced by the animals in the aquatic environment.

### Determination of anaerobic threshold (AT)

At 28 days of age, after being adapted to the liquid environment, the AT of the T group rats was determined by the minimum lactate test, as proposed for humans [23] and modified for rats [28,50]. This test consists of the following: a) a brief period of high intensity exercise to cause an increase in the circulating blood lactate, b) a brief period of recovery to ensure high levels of lactacidemia, c) exercise with progressive loads, increasing overhead and d) the collection of blood for the lactate analysis. Because the progressive portion of the lactate minimum test begins when the blood lactate concentrations are high, the test produces a characteristic progressive lactate profile in the form of a "U". The minimum blood lactate was defined as the intensity of exercise in which the "U curve", derived from the blood lactate values obtained during the test, reaches a minimum concentration and theoretically indicates the AT [23].

For the hyperlactacidemia induction, the animals were placed individually in tanks (100 × 80 × 80 cm) containing water at 31 ± 1°C, supporting high overloads relative to the body weight (bw) (20% at 28 days of age and 13% for adult rats) and allowed to swim for 30 seconds. After 30 seconds of rest, the rats were allowed to swim again with the same overloads until exhaustion. After a resting period (12 min for 28 day old rats and 9 min for adult rats), blood samples were collected by cutting the tail end to determine the lactate concentration, and the animals began the swimming exercise with progressively higher intensities [28,50]. The first load was equivalent to 5% bw at 28 days of age and 4% bw in adult rats, and the loads were increased by 1.0% bw in 28 day old rats and 0.5% bw in adult rats every 5 minutes until exhaustion of the animal. In each overload exchange, a blood sample was collected for lactate determination [28 modified by 50]. The blood lactate determination was performed by the enzymatic method [51].

Because the AT is often assumed to be characterized by high-intensity exercise in which the blood lactate does not increase more than 1 mmol/L from 10 to 30 min of exercise with constant overload [29], four days after the completion of the lactate minimum test, the rats were submitted to a single session of swimming supporting constant overloads corresponding to the lactate threshold (AT) that was determined by the test. Each test consisted of continuous swimming for 30 minutes with a 100% load with blood sampling in every 10 min to confirm the stabilization of lactate production.

### Training protocol

The T group was submitted to swimming exercise in individual tanks containing water at 31 ± 1°C for one hour per day, five days per week, for eight weeks (from 28 to 90 days of age). The rats supported overloads tied to the back with elastic (Flash elastic, São Paulo/SP, Brazil®), equivalent to 80% of the individual AT previously identified by the lactate minimum test.

### Insulin tolerance test (ITT)

At the end of the experiment, the insulin sensitivity was evaluated by the insulin tolerance test (ITT). The first blood sample was taken from the tail vein (time 0). Then, a solution of insulin at a dose of 150 mU/100 g of body weight was administered intraperitoneally. The blood samples were collected at 4, 8, 12 and 16 minutes post-insulin injection from heparinized capillaries and calibrated for 25 μl to determine the glucose concentrations using a commercial kit (Laborlab®). A single cut at the tail end was enough to collect all blood samples. The results were analyzed by calculating the removal rate of serum glucose (Kitt) [52]. The kitt was calculated by the formula (0.0693/t_1/2_) × 100 and expressed in %/min. The removal of blood glucose (t_1/2_) was calculated by curve least squares analysis of the blood glucose levels at the times of decay after the administration of insulin [53] using Microsoft Excel software 2007.

### Sacrifice of animals and obtaining biological material

#### Blood

At the end of the experiment, in the fed state and 48 hours after the last "in vivo" evaluation, the animals were sacrificed after anesthesia with CO_2_ in a 72 hour resting state, and the blood was collected for serum separation and determination of free fatty acids [54].

#### Liver

The liver was collected at time of sacrifice for weighing, and an aliquot (500 mg) was used to determine the concentration of triglycerides using a commercial kit (Laborlab®, Paulínia/SP, Brazil).

In a separate batch of animals (5/group), rats from both groups were sacrificed 60 minutes after receiving the intraperitoneal administration of 3 mCi ^3^H_2_O. Aliquots of the liver tissue were extracted and weighed to determine the lipogenic rate using tritiated water as previously described by Robinson & Williamson [55]. The lipid synthesis rate was expressed as μmol of ^3^H_2_O incorporated per hour per gram of hepatic tissue.

### Statistical analysis

The results were expressed as the means ± standard deviation. The Student t-test for independent samples was used to compare the mean body weight, rate of removal of serum glucose (Kitt), concentrations of FFA and triglycerides and hepatic lipogenic rate at the end of the experiment. The level of significance was preset at p < 0.05.

## Abbreviations

NAFLD: Nonalcoholic fatty liver disease; FFA: Free fatty acids; TG: Triglycerides; TNF-α: Tumor necrosis factor alpha; AT: Anaerobic threshold; bw: Body weight; ITT: Insulin tolerance test; BLT: Blood lactate threshold; MLSS: Maximum lactate steady state; EPOC: Excess post-exercise oxygen consumption; FA: Fatty acids; SREBP: Sterol regulatory element-binding proteins; FAS: Fatty acid synthase; ACC: Acetyl-CoA carboxylase; SCD1: Stearoyl-CoA Desaturase1; TLRs: Toll-like receptors

## Competing interests

The authors declare that they have no competing interests.

## Authors’ contributions

All of the authors contributed to the study, with regard to not only sample collection but also the preparation of this manuscript. All of the authors have read and approved the final version of this manuscript.
